# Gender differences in the subjective assessment of emotional state in Russians

**DOI:** 10.1192/j.eurpsy.2021.1604

**Published:** 2021-08-13

**Authors:** R. Shilko, L. Shaigerova, A. Dolgikh, O. Almazova, M. Rabeson

**Affiliations:** Faculty Of Psychology, Lomonosov Moscow State University, Moscow, Russian Federation

**Keywords:** mental health, Gender, emotional state, subjective assessment

## Abstract

**Introduction:**

Research into sociocultural mediation of mental health engages the factor of gender differences in the subjective assessment of emotional state.

**Objectives:**

The current study aims to identify the features of the subjective assessment of emotional state in men and women.

**Methods:**

The study involved 210 men and 403 women aged 14 to 76 years (M = 26.9; SD = 13.7) from six regions of the Russian Federation: Moscow, St. Petersburg, Udmurtia, Sakha, Sverdlovsk and Kemerovo. Participants were asked to evaluate their emotional state at the present time, choosing one of the following answers: “excellent”, “good”, “average”, “poor”, “very poor”.

**Results:**

The same pattern of the answer frequency distribution was established for men and women. “Good” was the most frequent answer (40% in men and 40% in women), followed by “average” (32% and 36%), “excellent” (18% and 12%), “poor” (9% and 11%), and “very poor” (1% and 2%). Statistical analysis on a rank scale with t-test for independent samples showed that the differences in the subjective assessment of emotional state in men and women are significant (t = 2.132; p = 0.033).

**Conclusions:**

Thus, both men and women rarely choose the extreme answers to assess their emotional state. Despite this similarity, there are statistically significant gender differences in the subjective assessments of emotional state: men are more likely to choose the answer “excellent” and use the answers “average”, “poor” and “very poor” less frequently. The reported study was funded by the RFBR, project number 17-29-02506.

